# Effects of motor imagery in recovery of nerve blockade in patients undergoing total knee replacement under spinal anesthesia: a randomized prospective controlled study

**DOI:** 10.1186/s13018-025-05936-4

**Published:** 2025-06-12

**Authors:** Sung Woo Hyung, Jeong Won Moon, Eun Sang Lee, Se Eun Jeong, Ji Young Min

**Affiliations:** 1 Department of Anesthesiology and Pain Medicine, Eunpyeong St. Mary’s Hospital, College of Medicine, The Catholic University of Korea, 1021 Tongil-ro, Eunpyeong-gu, Seoul, 03312 Republic of Korea; 2 Nursing Department of Eunpyeong St. Mary’s Hospital, College of Medicine, The Catholic University of Korea, Seoul, Republic of Korea

**Keywords:** Anesthesia, spinal, Motor imagery, Recovery of function, Postoperative period

## Abstract

**Background:**

Spinal anesthesia (SA) is widely used for total knee arthroplasty (TKA) due to its advantages in postoperative pain control and reduced opioid use. However, prolonged motor and sensory blockade remains a concern, delaying recovery and increasing patient discomfort. Given the limitations of current pharmacologic and physical therapy strategies in accelerating neural recovery, motor imagery (MI)—a cognitive technique that activates motor and sensory pathways without physical movement—has emerged as a potential neuromodulatory intervention. This study aimed to evaluate the effects of MI combined with active ankle range of motion (ROM) exercises on neural recovery after SA.

**Methods:**

In this prospective, randomized controlled trial, 76 female patients undergoing TKA under SA were enrolled and allocated to either an MI group (*n* = 40), who performed structured MI-ROM exercises during their post-anesthesia care unit (PACU) stay, or a non-MI group (*n* = 36), who received guided relaxation. Motor and sensory recovery were assessed at 30, 60, and 90 min postoperatively using the modified Bromage scale, pinprick test, and alcohol cotton test.

**Results:**

The MI group demonstrated significantly faster motor recovery at all time points (*P* = 0.002) and superior sensory recovery via pinprick testing at 60 and 90 min (*P* = 0.003), while no significant difference was found in alcohol cotton test results (*P* = 0.314).

**Conclusion:**

These findings suggest that MI is a feasible, non-invasive intervention that may enhance early motor and sensory recovery after SA, supporting its potential role in improving postoperative outcomes in TKA patients.

**Trial registration:**

Clinical Research Information Service (CRIS), KCT0010364, registered on March 28, 2025; retrospectively registered.

## Introduction

Spinal anesthesia (SA) is widely utilized in total knee arthroplasty (TKA) due to its favorable profile in postoperative pain control and reduced opioid use [1]. However, prolonged motor and sensory blockade following SA remains a notable concern, particularly in elderly patients, as it may delay mobilization, prolong post-anesthesia care unit (PACU) stay, and increase postoperative discomfort [2, 3]. A recent study on outpatient total joint arthroplasty reported a 52-minute longer median PACU stay in the neuraxial anesthesia group compared to the general anesthesia (GA) group [4]. Such delays in neural recovery can postpone the initiation of rehabilitation efforts, thereby impacting early mobilization and functional outcomes. These observations underscore the need for adjunctive strategies that facilitate faster neural recovery and support early functional restoration in TKA patients.

Accordingly, non-pharmacologic interventions that leverage neuroplastic mechanisms have gained interest. One such approach is motor imagery (MI), a cognitive strategy involving the mental rehearsal of movement without physical execution, which engages sensorimotor cortical circuits and has been shown to promote cortical reorganization [5]. MI has demonstrated benefits in enhancing motor recovery and sensorimotor integration across various neurological conditions, including stroke and Parkinson’s disease [6, 7]. By activating both motor and sensory pathways, MI may counteract SA-induced transient neural suppression, thereby supporting early mobilization and reducing the risk of delayed rehabilitation [8, 9].

A unique advantage of SA is the preservation of consciousness, which allows for the immediate postoperative application of MI-based cognitive neuromodulation. While MI has been extensively studied in the field of neurorehabilitation, its potential role in mitigating temporary SA-related motor and sensory blockade remains underexplored [10, 11]. Exploring MI in this context may offer a clinically feasible and low-risk strategy to enhance neural recovery and improve functional outcomes following orthopedic surgery.

Based on this rationale, this study hypothesized that early implementation of MI during postoperative period may promote motor and sensory recovery in TKA patients under SA. This study aimed to assess preliminary efficacy of MI as a behavioral neuromodulatory intervention to support early-phase functional recovery and inform evidence-based approaches in postoperative patient’s quality of recovery.

## Methods

### Study design and population

This prospective, randomized controlled trial was conducted at a single medical hospital (Eunpyeong St. Mary’s Hospital, College of Medicine, The Catholic University of Korea) between March 2022 and October 2022, in compliance with the tenets of the Declaration of Helsinki and the principles of Good Clinical Practice (GCP). Ethical approval was obtained from the Institutional Review Board and Hospital Research Ethics Committee of The Catholic University of Korea, Eunpyeong St. Mary’s Hospital (IRB protocol no. PC22OISI0032). The study was retrospectively registered with the Clinical Research Information Service (CRIS; https://cris.nih.go.kr) under the identifier KCT0010364 on March 28, 2025; the first patient was enrolled on May 16, 2022. Although retrospective registration limits optimal transparency according to ICMJE guidelines, the study complied with all relevant ethical and institutional guidelines and regulations. Written informed consent was obtained from all participants prior to enrollment.

### Inclusion and exclusion criteria

Female patients undergoing unilateral TKA under SA were eligible for enrollment if they met the following criteria: aged 50–75 years and classified as American Society of Anesthesiologists (ASA) physical status I–II. TKA is more commonly performed in females, who undergo the procedure at approximately two to three times the rate of males, with 60–75% of recipients being female. This trend is observed globally, including in South Korea, where the prevalence of degenerative knee osteoarthritis is significantly higher in females over the age of 50, contributing to increased TKA demand [12, 13]. Accordingly, this study included female patients to ensure a homogeneous study population and enhance the applicability of the findings. Exclusion criteria included patients with cognitive impairment, as MI requires active mental engagement, and those with included severe sensory neuropathy, moderate to severe depression or anxiety, significant visual or auditory impairment, prior TKA on the same knee or severe contralateral joint disease, chronic opioid use or central nerve system (CNS)-acting medications, and persistent or unexplained neurological symptoms. These conditions could impair MI engagement, alter sensory-motor processing, or affect rehabilitation outcomes, ensuring a more reliable assessment of MI’s effects on SA recovery.

### Randomization and blinding

Patients were randomly assigned to the MI or non-MI group in a 1:1 ratio using a pre-generated randomization list. Allocation concealment was ensured via sequentially numbered, opaque sealed envelopes opened immediately before enrollment. A double-blind design was implemented. Separate research assistants conducted pre- and post-intervention assessments to prevent detection bias, and all clinical staff—including the anesthesiologist, PACU nurses, rehabilitation providers, and ward personnel—remained blinded to group assignment to minimize performance bias. To equalize cognitive engagement and control for expectancy effects, the non-MI group received a time-matched structured educational session. Additionally, data analysts were blinded to group allocation during statistical analysis to ensure objectivity.

### Study protocol

**Fig. 1:**
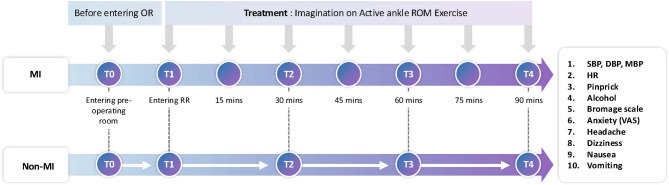
Study protocol. Schematic illustration of the study timeline and assessments performed at predefined time points. Clinical variables, including motor and sensory recovery, were evaluated at the following intervals. T1, immediately upon entering the post-anesthesia care unit (PACU); T2, 30 min after entering the PACU; T3, 60 min after entering the PACU; T4, 90 min after entering the PACU. Participants in the motor imagery (MI) group performed MI combined with active ankle range of motion (ROM) exercises during the PACU stay, whereas the control group received guided relaxation. VAS, visual analog scale (pain assessment tool); PACU, post-anesthesia care unit; ROM, range of motion; MI, motor imagery

The study protocol is outlined in Fig. 1. Upon arrival at the preoperative waiting area, patients in the MI group received a five-minute structured education session on active ankle range of motion (ROM) exercises incorporating MI. The training, conducted by the researcher using a standardized booklet, ensured comprehension and adherence to the intervention protocol. Similarly, the non-MI group received a structured educational session of equivalent duration, focusing on general postoperative recovery guidance rather than MI-related exercises. SA was induced and maintained according to institutional protocols, with patients positioned in the lateral decubitus position on the day of surgery. A lumbar puncture was performed at the L3-L4 or L4-L5 interspace using a 25G needle, and 10–12 mg of hyperbaric bupivacaine (Marcain Heavy 0.5%) was administered. Sensory blockade to at least the T10 dermatome was confirmed before surgical incision. No sedation was administered during the procedure. Following surgery, patients were transferred to the PACU, where those in the MI group engaged in MI combined with active ankle ROM exercises throughout their PACU stay [14]. The intervention was performed seven times at 15-minute intervals, beginning immediately upon PACU admission and continuing at 15, 30, 45, 60, and 75 min postoperatively, with the final session at 90 min. Each session lasted approximately one minute, with each movement repeated twice per session.

The structured MI- active ankle ROM intervention included:

1. Ankle dorsiflexion and plantarflexion - Patients performed repetitive movements by pulling the foot upward (dorsiflexion) and pointing it downward (plantarflexion) while lying in the supine position.
2. Ankle inversion and eversion - Patients alternated between moving the sole of the foot inward (inversion) and outward (eversion).
3. Circular foot and toe movements - Patients performed circular movements of the foot while simultaneously moving the toes.

In contrast, the non-MI group received guided breathing exercises and relaxation techniques to ensure comparable levels of cognitive engagement and attention. These sessions followed the same frequency and structured format as the MI- active ankle ROM intervention, with seven one-minute sessions performed at 15-minute intervals throughout the PACU stay. Each session lasted approximately one minute, with each breathing or relaxation technique repeated twice per session to match the repetition structure of the MI- active ankle ROM intervention.

### Clinical variables

Motor function, sensory blockade level, postoperative symptoms, anxiety levels, and physiological parameters were systematically assessed using validated measurement tools. Motor blockade was evaluated using the modified Bromage scale, which quantifies motor function on a 0–4 scale, with higher scores indicating greater motor blockade: 0 (able to lift the leg with the knee extended), 1 (unable to lift the leg but able to flex the knee), 2 (unable to flex the knee but able to flex the ankle), 3 (unable to flex the ankle but able to move the toes), and 4 (complete paralysis with no toe movement). Sensory blockade level was assessed using the pinprick test and alcohol cotton test. The pinprick test was performed using Touch Test Sensory Evaluators (NC12775-14, North Coast Medical, USA), applying a fine needle stimulus along the left mid-axillary line, with the highest dermatome level at which the patient failed to perceive pain recorded as the sensory blockade level. The alcohol cotton test assessed cold sensation loss along the same mid-axillary line, with the highest dermatome level of cold sensation loss recorded as the cold sensory blockade level. The spinal blockade level was numerically quantified by assigning segmental values: thoracic (T1–T12) = 1–12, lumbar (L1–L5) = 13–17. Anxiety levels were measured using the Visual Analog Scale (VAS), developed by Cline, Herman, Shaw, and Morton, in which participants marked their perceived anxiety level on a 100 mm horizontal scale (0 = no anxiety, 10 = extreme anxiety), with higher scores indicating greater anxiety. Postoperative symptoms, including headache, dizziness, nausea, and vomiting, were assessed through direct patient questioning and recorded accordingly. Hemodynamic parameters, including systolic blood pressure (SBP), diastolic blood pressure (DBP), mean blood pressure (MBP), and heart rate (HR), were continuously monitored using a patient monitoring system (MX450 Monitor, Philips Medical System, USA) while the patient remained in the supine position, ensuring hemodynamic stability during post-anesthesia recovery.

### Data collection

In the PACU, assessments were conducted for both the MI and non-MI groups at four time points: upon arrival and at 30-, 60-, and 90-minutes post-admission. At each time point, motor and sensory nerve blockade levels were evaluated using the modified Bromage scale, pinprick test, and alcohol cotton test to monitor changes over time. Anxiety levels were reassessed using the VAS, and postoperative symptoms (headache, dizziness, nausea, and vomiting) were recorded based on direct patient questioning. Additionally, physiological parameters—including non-invasive blood pressure (NIBP) and HR—were recorded at each assessment point.

### Primary and secondary outcomes

The primary outcome of this study was to compare differences in motor and sensory nerve recovery between the two groups. The secondary outcomes included assessing differences in physiological symptoms such as anxiety, headache, dizziness, nausea, and vomiting between the two groups.

### Sample size calculation and assumptions

The sample size estimation was based on a previous study investigating the therapeutic role of motor MI during the acute phase after TKA [15]. In that study, the MI group demonstrated greater functional recovery (83.99% ± 6.75, 95% confidence interval [C.I] = 3.02%) than the control group (79.61% ± 6.75, 95% C.I = 3.02%), with a relative improvement of 4.37% ± 2.1% from pre-test to post-test. Given the physiological similarities of MI after motor recovery following TKA and SA, the expected difference in the modified Bromage scale was assumed to be comparable. Based on this assumption, a two-tailed power analysis was conducted with an alpha level of 0.05 and a power of 0.80 (β = 0.20). The estimated effect size (Cohen’s d) was calculated based on the mean difference 4.37% and standard deviation (SD) 6.75% reported in the previous study, yielding an effect size of 0.647. Using these parameters, the required sample size per group was determined to be 38 participants. To account for an anticipated 10% dropout rate, the final recruitment target was set at 41 participants per group.

### Statistical analysis

Missing data were excluded from the analysis. Continuous variables are presented as mean (standard deviation), while categorical variables are expressed as number (percentage). Categorical variables were analyzed using the chi-squared test or Fisher’s exact test, as appropriate, with group homogeneity assessed using the chi-squared test (χ² test). Repeated measures analysis of variance (Repeated Measure ANOVA) was performed to evaluate changes over time in hemodynamic parameters, modified Bromage scale, sensory recovery levels assessed by the pinprick and alcohol cotton tests, and anxiety levels. To further examine specific changes across measurement points, post-hoc analysis using Bonferroni correction was conducted. The incidence of postoperative symptoms, including headache, nausea, vomiting, and dizziness, was analyzed using the chi-squared test (χ² test). Values with *P* < 0.05 were considered statistically significant.

## Results

### Patients’ demographics

**Fig. 2:**
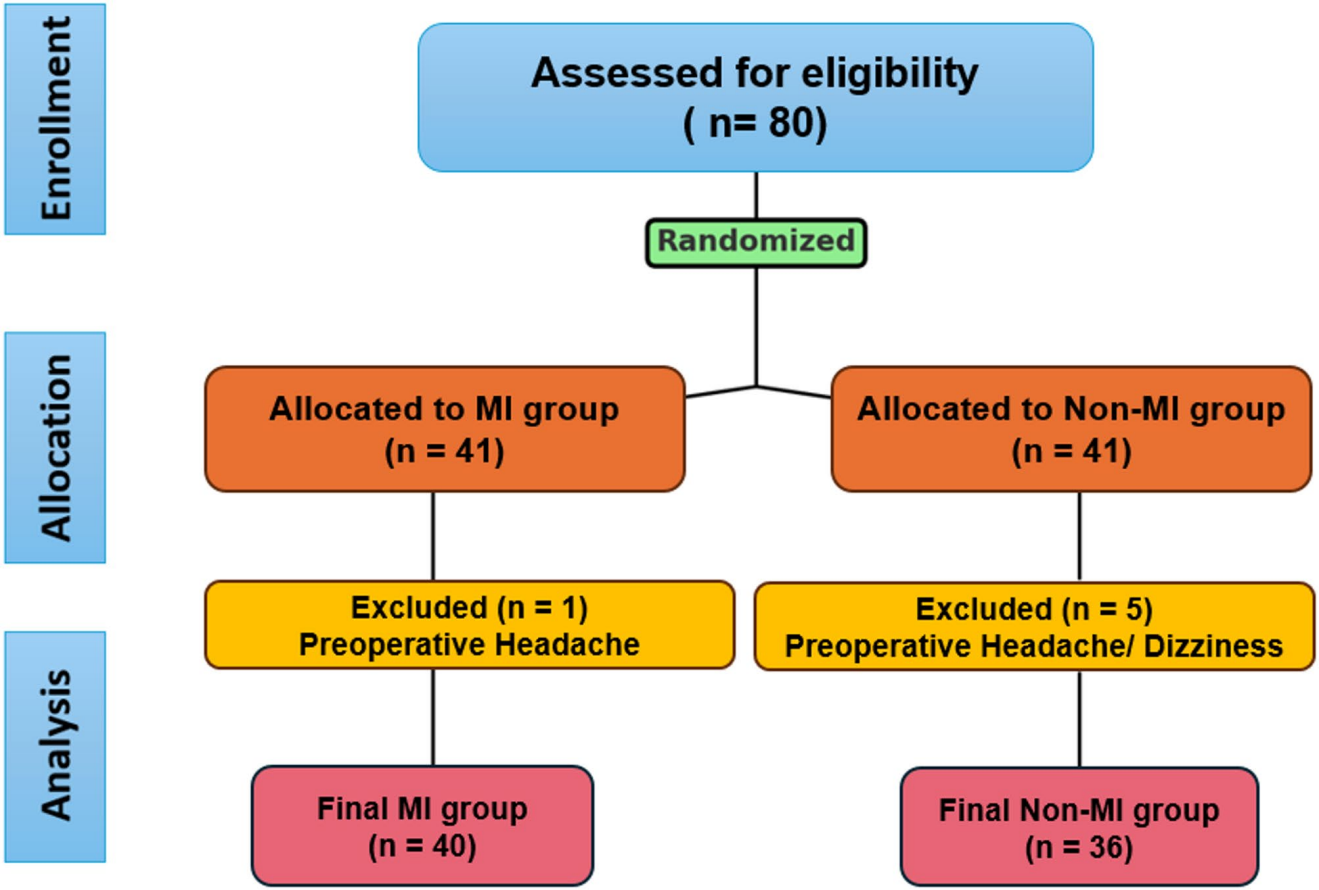
Consolidated standard of reporting trial (Consort) flow diagram

Of the 82 female patients initially enrolled, five were excluded due to preoperative symptoms, resulting in a final sample of 40 patients in the MI group and 36 in the non-MI group (Fig. 2). Baseline parameters measured in the preoperative room, including motor blockade level (modified Bromage scale), sensory blockade levels (pinprick and alcohol cotton tests), and physiologic symptom (headache, dizziness, nausea, vomiting, anxiety), showed no significant differences between the two groups (Table 1). Additionally, at entering PACU, no significant differences between groups were observed (Table 2). Hemodynamic stability was maintained intraoperatively without complications, and both groups completed the perioperative course uneventfully, with no adverse events during recovery.

**Table 1:** Baseline demographic and clinical characteristics of patients in the motor imagery (MI) and Non MI groups

	Subgroup	MI group (*n* = 40)	Non-MI group (*n* = 36)	*P*
Age, years		66.1 (5.8)	67.2 (5.9)	0.107
Height, cm		154.8 (7.1)	153.5 (6.9)	0.425
Weight, kg		66.2 (8.9)	64.1 (9.2)	0.094
BMI, kg/m^2^		26.4 (3.5)	25.7 (3.8)	0.191
ASA	1	3 (7.5)	3 (8.3)	0.018
	2	37 (92.5)	33 (91.7)	
Anesthesia duration, min		72.5 (3.5)	73.0 (2.8)	0.891
***Preoperative room***				
*Hemodynamic parameters*				
SBP, mmHg		152.1 (15.9)	151.6 (19.6)	0.690
DBP, mmHg		86.6 (10.1)	83.0 (13.2)	0.145
MBP, mmHg		108.4 (10.4)	105.9 (14.0)	0.261
HR, beats/ min		71.5 (12.3)	70.3 (10.9)	0.715
*Motor blockade level*				
Modified Bromage scale		0.0 (0.0)	0.0 (0.0)	1.000
*Sensory blockade level*				
Pain (pinprick test)		17.0 (0.0)	17.0 (0.0)	1.000
Temperature (alcohol cotton test)		17.0 (0.0)	17.0 (0.0)	1.000
*Physiologic symptoms*				
Headache		0 (0.0)	0 (0.0)	1.000
Dizziness		0 (0.0)	0 (0.0)	1.000
Nausea		0 (0.0)	0 (0.0)	1.000
Vomiting		0 (0.0)	0 (0.0)	1.000
Anxiety, VAS		2.1 (2.8)	1.3 (2.1)	0.066

Sex, ASA and physiologic symptom are presented as number (percentage); other variables are shown as mean (standard deviation). Anesthesia duration is defined as the time elapsed from the initiation of spinal anesthesia to the arrival in the post-anesthetic care unit. Modified Bromage scale quantifies motor function on a 0–4 scale. The sensory recovery level of pain (pinprick test) and temperature (alcohol test) are numerically quantified using segmental values: thoracic (T1–T12) = 1–12, lumbar (L1–L5) = 13–1*7. MI*, motor imagery; *BMI*, body mass index; *ASA*, American Society of Anesthesiologists physical status classification; *SBP*, systolic blood pressure; *DBP*, diastolic blood pressure; *MBP*, mean blood pressure; *HR*, heart rate; *VAS*, visual analog scale. * *P* < 0.05

**Table 2:** Comparison of hemodynamic parameters and recovery profiles between the motor imagery (MI) and Non-MI groups in the post anesthesia care unit (PACU)

	Entering PACU	30 min post-PACU entry	60 min post-PACU entry	90 min post-PACU entry	P__Group*Time_
SBP (mmHg)					
MI (*n* = 40)	118.7 (13.1)	129.3 (16.1)^†^	127.5 (17.7)^†^	126.4 (16.8)^†^	0.468
Non-MI (*n* = 36)	116.0 (23.8)	131.4 (16.7)^†^	131.9 (16.3)^†^	126.9 (15.3)^†^	
DBP (mmHg)					
MI (*n* = 40)	74.7 (9.3)	78.7 (11.0)	76.7 (10.5)	78.5 (10.7)	0.644
Non-MI (*n* = 36)	75.2 (13.3)	76.4 (10.9)	75.0 (9.0)	76.1 (11.3)	
MBP (mmHg)					
MI (*n* = 40)	89.3 (9.0)	95.5 (11.0)^†^	93.6 (10.8)^†^	94.4 (10.4)^†^	0.811
Non-MI (*n* = 36)	88.8 (14.8)	94.7 (10.6)^†^	94.0 (9.3)^†^	93.1 (10.6)^†^	
HR (beats/min)					
MI (*n* = 40)	62.9 (9.5)	61.8 (10.1)^†^	62.7 (12.5)^†^	63.7 (12.4)	0.099
Non-MI (*n* = 36)	66.8 (10.4)	62.6 (10.9)^†^	61.5 (8.5)†	64.0 (9.4)	
Motor recovery level					
**Modified Bromage scale**					
**MI (*n* = 40)**	2.9 (1.7)	**1.5 (1.6)** ^*,†^	**0.9 (1.8)** ^*,†^	**0.5 (1.1)** ^*,†^	**0.002** ^**^
**Non-MI (*n* = 36)**	3.4 (0.9)	**2.9 (1.6)** ^**†**^	**1.7 (1.6)** ^**†**^	**0.8 (1.2)** ^**†**^	
**Sensory recovery level**					
**Pain: pinprick test**					
**MI (*n* = 40)**	10.0 (3.2)	11.9 (2.9)^†^	**14.0 (2.7)** ^*, †^	**15.1 (2.2)** ^*,†^	**0.003** ^**^
**Non-MI (*n* = 36)**	9.6 (1.7)	10.8 (2.3)^†^	**12.3 (2.2)** ^**†**^	**13.2 (1.8)** ^**†**^	
Temperature: alcohol cotton test					
MI (*n* = 40)	8.7 (2.9)	10.2 (3.0)^†^	11.7 (2.7)^†^	13.2 (2.8)^†^	0.314
Non-MI (*n* = 36)	8.2 (2.1)	10.1 (2.3)^†^	11.3 (2.4)^†^	12.1 (2.1)^†^	

All variables are shown as mean (standard deviation)

Modified Bromage scale quantifies motor function on a 0–4 scale. The sensory recovery level of pain (pinprick test) and temperature (alcohol test) are numerically quantified using segmental values: thoracic (T1–T12) = 1–12, lumbar (L1–L5) = 13–17

*MI*, motor imagery; *PACU*, post-anesthetic care unit; *SBP*, systolic blood pressure; *DBP*, diastolic blood pressure; *MBP*, mean blood pressure; *HR*, heart rate

*Entering PACU*, when entering the PACU; *30 min post-PACU entry*, 30 min after entering the PACU; *60 min post-PACU entry*, 60 min after entering the PACU; *90 min post-PACU entry*, 90 min after entering the PACU

^**^P __Group×*Time_, *P* value of the group, and time interaction obtained by repeated measure ANOVA ^**^P < 0.05

^*^Bonferroni-corrected *P* < 0.05 compared with the non-MI group

^†^Bonferroni-corrected *P* < 0.05 compared with the value of “Entering PACU” in each group

### Hemodynamic variables in PACU

Changes in hemodynamic parameters over time are presented in Table 2. Upon arrival in the PACU, SBP and MBP were comparable between the MI and non-MI groups. Both groups exhibited a transient increase in SBP and MBP at 30 min post-PACU entry; however, these values remained within the normal range and did not require antihypertensive intervention. Additionally, there were no significant differences between the groups, with no interaction effect between time and group (*P*__Group*Time_ = 0.468 and 0.811, respectively). DBP and HR remained stable throughout the PACU stay, with no significant differences between the groups (*P*__Group*Time_ = 0.644 and 0.099, respectively).

### Recovery of motor and sensory block in PACU

**Fig. 3:**
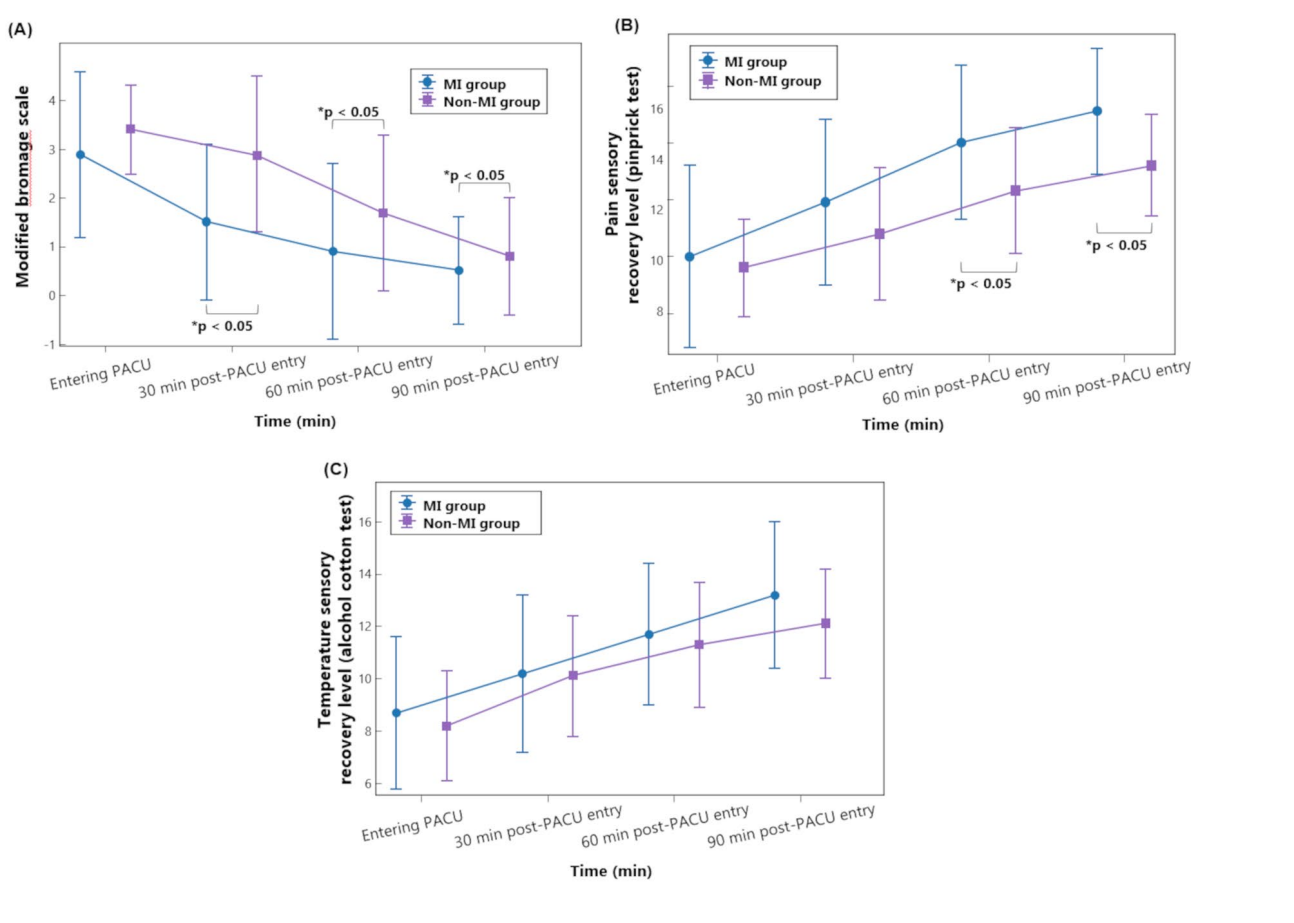
Comparison of postoperative recovery trends between the motor imagery (MI) and non-MI groups in the post-anesthesia care unit (PACU). Recovery trends are illustrated for the initial 90 min after PACU entry. Data are presented as mean ± 95% Confidence interval (C.I). The MI group is indicated in blue, and the non-MI (control) group in purple. (**A**) Motor recovery, assessed by the modified Bromage scale, which measures motor blockade based on patients’ ability to perform movements. (**B**) Pain sensory recovery, evaluated by the pinprick test, which assesses the return of pain sensation across dermatomes. (**C**) Temperature sensory recovery, assessed using an alcohol cotton test, which evaluates recovery of cold sensation across dermatomes. For sensory assessments (pain and temperature), the spinal blockade levels were numerically quantified according to spinal segments: thoracic segments (T1–T12) assigned values 1–12, and lumbar segments (L1–L5) assigned values 13–17. MI, motor imagery; PACU; post-anesthetic care unit. Entering PACU, when entering the PACU; 30 min post-PACU entry, 30 min after entering the PACU; 60 min post-PACU entry, 60 min after entering the PACU; 90 min post-PACU entry, 90 min after entering the PACU

Upon arrival in the PACU, there were no statistically significant differences in motor and sensory blockade levels between the two groups. In motor blockade recovery, the modified Bromage scale scores significantly decreased over time in both groups (Bonferroni-corrected *P* < 0.05 for all comparisons). However, the rate of decrease differed significantly between groups, with the MI group demonstrating a more rapid reduction in modified Bromage scale (Fig. 3-A). The MI group exhibited significantly lower scores at 30, 60, and 90 min post-PACU entry compared to the non-MI group (*P*__Group*Time_ = 0.002, partial η² = 0.065). Sensory blockade recovery, as assessed by the pinprick test, also showed a significant increase over time in both groups (Bonferroni-corrected *P* < 0.05, Fig. 3-B). The MI group exhibited significantly higher recovery level at 60, and 90 min post-PACU entry compared to the non-MI group (*P*__Group*Time_ = 0.003, partial η² = 0.059). The alcohol cotton test results followed a similar trend, showing a gradual decrease in sensory blockade over time (Fig. 3-C). However, no statistically significant difference was observed between the groups (*P*__Group*Time_ = 0.314). Changes in the recovery profile of nerve blockade over time are presented in Table 2.

### Differences in physiologic symptoms and anxiety

Differences in physiological symptoms and anxiety are presented in Table 3. The incidence of headache, dizziness, nausea, and vomiting was low in both groups, with no significant differences (*P* = 1.000 for all comparisons). Headache occurred in one patient (2.5%) in the MI group at 60 min post-PACU entry, while dizziness was reported in two patients (5.0%) at 30 min post-PACU entry, with no cases in the non-MI group. Nausea was observed at 30, 60, and 90 min post-PACU entry in the MI group but was absent in the non-MI group. Vomiting occurred in two MI patients (2.5%) at 30 and 90 min post-PACU entry, with no cases in the non-MI group. Anxiety levels (VAS) gradually decreased in both groups. While there was a significant difference between the two groups at 30 and 60 min after admission to the recovery room, no significant difference was observed at 90 min.

**Table 3:** Incidence of adverse effects and anxiety scores between the motor imagery (MI) and non-MI groups in the post anesthesia care unit (PACU)

	Entering PACU	30 min post-PACU entry	60 min post-PACU entry	90 min post-PACU entry	P__Group*Time_
Headache					
MI (*n* = 40)	0 (0.0)	0 (0.0)	1 (2.5)	0 (0.0)	1.000
Non-MI (*n* = 36)	0 (0.0)	0 (0.0)	0 (0.0)	0 (0.0)	
Dizziness					
MI (*n* = 40)	0 (0.0)	2 (5.0)	0 (0.0)	0 (0.0)	1.000
Non-MI (*n* = 36)	0 (0.0)	0 (0.0)	0 (0.0)	0 (0.0)	
Nausea					
MI (*n* = 40)	0 (0.0)	3 (7.5)	1 (2.5)	1 (2.5)	1.000
Non-MI (*n* = 36)	0 (0.0)	0 (0.0)	0 (0.0)	0 (0.0)	
Vomiting					
MI (*n* = 40)	0 (0.0)	1 (2.5)	0 (0.0)	1 (2.5)	1.000
Non-MI (*n* = 36)	0 (0.0)	0 (0.0)	0 (0.0)	0 (0.0)	
Anxiety (VAS)					
MI (*n* = 40)	0.6 (2.1)	0.2 (1.7) ^*^	0.2 (1.3) ^*^	0.2 (1.6)	0.099
Non-MI (*n* = 36)	0.9 (2.4)	0.8 (2.3)	0.4 (1.1)	0.2 (0.6)	

Physiologic symptoms are presented as number (percentage); Anxiety is shown as mean (standard deviation)

*MI*, motor imagery; *PACU;* post-anesthetic care unit; *VAS*, visual analog scale

*Entering PACU*, when entering the PACU; *30 min post-PACU entry*, 30 min after entering the PACU; *60 min post-PACU entry*, 60 min after entering the PACU; *90 min post-PACU entry*, 90 min after entering the PACU

^**^P __Group×*Time_, *P* value of the group, and time interaction obtained by repeated measure ANOVA ^**^P < 0.05

^*^Bonferroni-corrected *P* < 0.05 compared with the non-MI group

^†^Bonferroni-corrected *P* < 0.05 compared with the value of “Entering PACU” in each group

## Discussion

This study suggests that MI combined with active ankle ROM exercises may facilitate early neural recovery following SA, particularly in motor and sensory domains. Patients in the MI group exhibited significantly faster motor recovery as assessed by the modified Bromage scale, demonstrating lower scores at multiple postoperative time points (30 min post-PACU entry: 1.5 ± 1.6 vs. 2.9 ± 1.6; 60 min post-PACU entry: 0.9 ± 1.8 vs. 1.7 ± 1.6; 90 min post-PACU entry: 0.5 ± 1.1 vs. 0.8 ± 1.2; *P*__Group*Time_ = 0.002) compared to those receiving guided relaxation. These findings support the feasibility of MI as a cognitive strategy during the immediate postoperative period—a phase characterized by restricted voluntary movement yet preserved central nervous system responsiveness to internally generated motor activity. However, the primary outcome measure used in this study, the modified Bromage scale, specifically assesses lower limb motor blockade rather than broader functional outcomes such as mobility, balance, or activities of daily living. Therefore, interpretations regarding overall functional recovery based solely on these findings should be made cautiously.

Unlike prior applications of MI in orthopedic or neurological rehabilitation—which typically begin during later recovery stages, after anesthetic effects have fully resolved or within chronic rehabilitation settings—this study uniquely introduced MI during the immediate postoperative period in the PACU. This immediate postoperative context is clinically distinct, characterized specifically by residual SA-induced motor blockade, diminished peripheral sensory feedback, and transient disruptions in sensorimotor integration. Previous MI interventions primarily focused on optimizing functional outcomes during advanced recovery stages, emphasizing long-term neuroplastic adaptations and improvements in chronic motor performance rather than early neural reactivation following anesthesia-induced inhibition [7, 16, 17]. By contrast, this study explored MI implementation specifically during an acute timeframe when peripheral movement is still pharmacologically inhibited but central motor planning functions remain neurologically accessible. Under these conditions, internal rehearsal of movements through MI may stimulate cortical motor circuits despite peripheral immobilization, potentially supporting neural recovery at a clinically relevant time point. The findings from this study suggest that initiating MI during this early postoperative period can be associated with improved motor and sensory recovery outcomes, highlighting a previously underexplored yet potentially valuable period for cognitive motor interventions. Such improvements may be underpinned by specific neurophysiological adaptations within the central nervous system in response to reduced afferent input caused by SA.

The neurophysiological mechanisms underlying these observations may involve the central nervous system’s compensatory adaptation to reduced afferent input during SA. Peripheral sensory blockade associated with SA diminishes ascending sensory signals from the lower limbs, leading the central nervous system to rely more heavily on internally generated motor commands, thereby altering sensorimotor integration processes [18]. Such internal reliance during reduced peripheral input creates a unique environment suitable for interventions that stimulate endogenous motor activity. In this regard, MI, which involves internally rehearsing movements without physical execution, closely aligns with this adaptive mechanism by activating higher-order motor regions, including the primary motor cortex (M1), supplementary motor area (SMA), premotor cortex, and cerebellum. Indeed, previous neuroimaging and neurophysiological studies consistently demonstrate that internally mediated motor rehearsal enhances cortical activation and corticospinal excitability, thereby facilitating neural plasticity [19–22]. Furthermore, combining MI with peripheral stimulation could offer a synergistic effect, as MI reinforces endogenous motor planning at the cortical level, while peripheral stimulation simultaneously provides additional sensory inputs, collectively amplifying cortical and subcortical neuroplastic changes beyond either modality alone in the prior research [23]. Therefore, MI may help sustain endogenous motor network activity despite afferent suppression [24]. In support of this theoretical perspective, the observed earlier recovery of toe flexion in the MI group provides preliminary clinical evidence; however, the observational nature of the current study limits definitive conclusions, and alternative explanations or causal relationships cannot be excluded [25, 26].

Sensory recovery also appeared to benefit from MI. The MI group demonstrated significantly earlier recovery of pain sensation in the L3–S1 dermatomes, as evidenced by higher pinprick test scores at multiple postoperative time points (60 min post-PACU entry: 14.0 ± 2.7 vs. 12.3 ± 2.2; 90 min post-PACU entry: 15.1 ± 2.2 vs. 13.2 ± 1.8; *P*__Group×Time_ = 0.003) compared to those receiving guided relaxation. While MI’s impact on sensory pathways is less established than its motor effects [27, 28], existing evidence suggests that MI activates sensorimotor cortical networks—including the primary somatosensory cortex (S1) and SMA—and thus may facilitate the integration of sensory and motor information [29–32]. Enhanced somatosensory evoked potentials (SSEPs) observed during MI further support this hypothesis [33]. However, MI’s effect appeared to be modality-specific, as no significant difference in cold sensation recovery was noted between the groups, as indicated by similar alcohol cotton test scores across all postoperative time points (e.g., 30 min post-PACU entry: 10.2 ± 3.0 vs. 10.1 ± 2.3; 60 min post-PACU entry: 11.7 ± 2.7 vs. 11.3 ± 2.4; 90 min post-PACU entry: 13.2 ± 2.8 vs. 12.1 ± 2.1; *P*__Group×Time_ = 0.314), suggesting a limited effect of MI on thermoreceptive recovery. This observation aligns well with the established sequence of neural recovery following spinal anesthesia, wherein motor function typically recovers first, followed sequentially by tactile sensation, pain sensation, and finally temperature sensation [34]. Such differential recovery patterns can be explained by the distinct neurophysiological properties of nerve fibers involved. Pain sensation is primarily mediated by lightly myelinated Aδ fibers, whereas temperature (cold) sensation predominantly relies on unmyelinated C fibers [35, 36]. Prior studies have demonstrated that myelinated Aδ fibers recover more rapidly from anesthetic blockade compared to unmyelinated C fibers, due to differences in fiber diameter, myelination status, and anesthetic susceptibility [37]. Considering that MI preferentially engages cortical and subcortical sensorimotor pathways associated with discriminative sensory processing, it may specifically enhance recovery processes involving faster-conducting myelinated fibers (such as Aδ fibers), rather than those mediated by slower, unmyelinated fibers (such as C fibers). Therefore, the observed limited effect of MI on thermoreceptive pathways appears physiologically plausible.

Importantly, the observed enhancement in early neural recovery through MI may offer clinical benefits during the immediate postoperative period. If MI facilitates earlier resolution of spinal anesthesia-induced motor and sensory blockade, this could potentially lead to smoother emergence from anesthesia and earlier initiation of postoperative management such as mobilization and physical therapy. This accelerated neural recovery might subsequently reduce the time spent in the PACU, improving patient throughput and optimizing resource utilization. Shorter PACU stays may also potentially decrease certain complications, including respiratory issues or prolonged immobilization-related problems. The early motor and sensory recovery promoted by MI conceptually aligns with Enhanced Recovery After Surgery (ERAS) protocols, which emphasize rapid mobilization, reduced analgesic use, and accelerated functional recovery [38, 39]. However, the current study did not directly evaluate whether MI indeed shortens PACU stay or enhances practical implementation of ERAS protocols; thus, these benefits remain speculative. Further research is warranted to conclusively verify such potential outcomes. Additionally, as this study did not assess long-term outcomes, broader implications regarding the clinical benefits of accelerated neural recovery should be interpreted cautiously. Given that delays in mobilization could negatively influence postoperative complications and rehabilitation progress, further comprehensive and objective assessments are warranted to clarify the clinical effectiveness of interventions targeting early neurologic readiness. Although such benefits remain speculative at present, they could be particularly impactful for patients vulnerable to prolonged neuromuscular blockade, such as those undergoing TKA.

In this patient population, enhanced early neural recovery through MI could potentially contribute to improved postoperative outcomes. Patients undergoing TKA are particularly vulnerable to delayed neuromuscular recovery, which can lead to significant postoperative complications, including thromboembolism, pressure injury, and muscle deconditioning [40]. In this context, MI represents a low-risk, adjunctive intervention that may help activate central motor networks during the immediate postoperative period, prior to active mobilization. Improved early motor recovery—such as faster restoration of toe flexion and lower limb strength—can directly contribute to earlier mobilization, reduce the risk of muscle atrophy, and improve overall patient independence during early rehabilitation stages. Recent evidence further expands on this potential by demonstrating that MI, when combined with standard physiotherapy in the days following TKA, leads to enhanced functional performance and better preservation of quadriceps strength in the early postoperative phase [41]. Thus, taken together, these findings suggest that MI may positively influence the entire postoperative recovery continuum—from immediate resolution of anesthesia-induced neural blockade to subsequent functional rehabilitation. Similarly, the modest improvements observed in early sensory recovery—particularly pain sensation—may also have meaningful clinical implications within fast-track TKA protocols by enabling earlier initiation of mobilization and structured rehabilitation. Although subtle, these sensory improvements could contribute to patient comfort, reduce analgesic needs, and further support rapid recovery protocols. Future research is needed to clearly define the direct clinical outcomes associated with both motor and sensory recovery improvements, including their impacts on mobilization timelines, complication rates, patient comfort, analgesic consumption, and hospital length of stay. Moreover, evidence from neurologic rehabilitation highlights that MI may promote neuroplasticity and enhance functional recovery [42], supporting its application in orthopedic postoperative care. The MI protocol used in this study is brief, structured, and resource-efficient, making it potentially compatible with existing fast-track rehabilitation pathways. Despite these potential advantages, successful implementation into routine clinical practice, especially in busy PACU settings, may face practical challenges such as limited staff availability, the need for specialized training, and variations in patients’ cognitive readiness immediately after anesthesia. While advanced technology-based methods (e.g., mobile applications, sensor tracking, and neurofeedback systems) offer potential for delivering MI interventions, immediate practical application in the PACU may not be feasible due to these constraints. Alternatively, clinician-guided scripts or simple audiovisual materials could provide more practical and scalable solutions. Additionally, incorporating brief MI training into preoperative education may enhance patient familiarity and compliance, thus facilitating effective participation in MI during the immediate postoperative period. MI effectiveness may also vary by individual patient factors, including cognitive capacity, imagery vividness, and prior experience. Clearer delineation of these clinical outcomes—including mobilization timelines, complication rates, analgesic requirements, and patient comfort—would further substantiate the practical benefits of MI implementation.

This study has several limitations that should be considered when interpreting the findings. First, the single-center design and homogeneous patient population (50–75-year-old female patients) may limit the external validity of the results. Institutional differences in perioperative management and demographic variability could influence outcomes, potentially reducing the generalizability of MI’s effect [43]. Additionally, although limiting participants to females ensured homogeneity, it also restricts the generalizability to broader populations. Research indicates that neural and cognitive recovery patterns may differ between sexes, influenced by hormonal, neurophysiological, and psychosocial factors. For instance, studies have shown that women often exhibit greater pain sensitivity and different analgesic responses compared to men, potentially affecting postoperative recovery trajectories [44, 45]. Moreover, variations in motor function recovery and cognitive processing between genders have been documented, suggesting that sex-specific factors could influence the efficacy of MI interventions [46]. Subsequent multicenter trials involving more diverse populations, including younger and male patients, would clarify the impact of sex and age on responsiveness to MI. Second, the primary outcome of this study specifically assessed motor recovery through the modified Bromage scale, which measures lower limb motor blockade rather than comprehensive functional recovery. While the findings of this study support early neural recovery at a motor and sensory level, the modified Bromage scale does not encompass broader functional or patient-reported outcomes, such as gait, balance, or activities of daily living (ADLs). Thus, the clinical relevance of these improvements to overall postoperative functional status remains uncertain. Incorporating comprehensive functional and patient-reported outcomes in future investigations would provide greater clarity on the broader clinical implications of MI. Third, an important limitation of this study is that individual variability in MI proficiency—such as participant compliance and imagery vividness—was not objectively assessed. Although mental engagement likely influenced recovery trajectories, its degree and clinical significance remain uncertain due to the absence of standardized assessments [47, 48]. Consequently, this omission limits the interpretation of the findings regarding the precise impact of MI. Future research should incorporate standardized MI proficiency assessments, including neurophysiological monitoring (e.g., electroencephalography, functional MRI) or validated psychometric tools (e.g., Motor Imagery Questionnaire - Revised, Second Version), to quantify interindividual differences and refine MI implementation strategies. Fourth, sensory recovery assessments were based on subjective clinical tests, including pinprick and alcohol cotton test which are widely used but inherently subjective and prone to interobserver variability. While clinically practical, this subjectivity may limit the reliability and reproducibility of findings. Furthermore, although the pinprick test assesses recovery of pain sensation at a sensory-discriminative level, it does not capture subjective experiences of pain intensity, discomfort, or analgesic requirements. Thus, comprehensive assessment of postoperative pain was not included, restricting interpretations of patients’ subjective postoperative experiences and their potential impact on MI engagement and recovery. Incorporating objective neurophysiological measures, such as somatosensory evoked potentials (SSEPs) or quantitative sensory testing (QST), could enhance accuracy and provide mechanistic insights into sensory recovery processes [49]. Finally, the observation window was limited to the first 90 min postoperatively. Although this early phase is critical for mobilization and PACU discharge, the durability of MI’s benefits over the full rehabilitation course remains unknown. Longitudinal assessments over extended postoperative periods would help elucidate the long-term impact of MI on clinically relevant outcomes, including walking ability, rehabilitation progress, and overall recovery quality.

## Conclusion

In conclusion, this study provides preliminary evidence that structured MI, when implemented during early postoperative care, may facilitate motor and sensory recovery following SA in patients undergoing TKA. MI may complement traditional orthopedic rehabilitation protocols and enhance recovery trajectories in high-risk surgical populations. Further research is warranted to validate these findings in larger, diverse cohorts and to assess the durability of MI’s benefits throughout the rehabilitation continuum.

## Data Availability

The datasets used and analysed in the current study are available from the corresponding author upon reasonable request.

## Abbreviations

ASA: American Society of Anesthesiologists
BMI: Body Mass Index
CNS: Central Nervous System
CRIS: Clinical Research Information Service
DBP: Diastolic Blood Pressure
EEG: Electroencephalography
fMRI: Functional Magnetic Resonance Imaging
GCP: Good Clinical Practice
HR: Heart Rate
IRB: Institutional Review Board
MBP: Mean Blood Pressure
M1: Primary Motor Cortex
MI: Motor Imagery
MIQ-RS: Motor Imagery Questionnaire– Revised, Second Version
NIBP: Non-Invasive Blood Pressure
PACU: Post-Anesthesia Care Unit
QST: Quantitative Sensory Testing
ROM: Range of Motion
SA: Spinal Anesthesia
SBP: Systolic Blood Pressure
SMA: Supplementary Motor Area
SSEP: Somatosensory Evoked Potential
TKA: Total Knee Arthroplasty
VAS: Visual Analog Scale
